# Impact of Flavonols on Cardiometabolic Biomarkers: A Meta-Analysis of Randomized Controlled Human Trials to Explore the Role of Inter-Individual Variability

**DOI:** 10.3390/nu9020117

**Published:** 2017-02-09

**Authors:** Regina Menezes, Ana Rodriguez-Mateos, Antonia Kaltsatou, Antonio González-Sarrías, Arno Greyling, Christoforos Giannaki, Cristina Andres-Lacueva, Dragan Milenkovic, Eileen R. Gibney, Julie Dumont, Manuel Schär, Mar Garcia-Aloy, Susana Alejandra Palma-Duran, Tatjana Ruskovska, Viktorija Maksimova, Emilie Combet, Paula Pinto

**Affiliations:** 1 iBET/ITQB, Molecular Nutrition & Health Laboratory, 2780-157 Oeiras, Portugal; rmenezes@ibet.pt; 2 Division of Diabetes and Nutritional Sciences, Faculty of Life Sciences and Medicine, King’s College London, London SE1 9NH, UK; ana.rodriguez-mateos@kcl.ac.uk; 3 FAME Laboratory, School of Exercise Science, University of Thessaly, 42100 Volos, Greece; akaltsat@gmail.com; 4 CEBAS-CSIC, E-30100 Murcia, Spain; agsarrias@cebas.csic.es; 5 Unilever R&D, 3133AT Vlaardingen, The Netherlands; Arno.Greyling@unilever.com; 6 University of Nicosia, CY1700 Engomi, Cyprus; giannaki.c@unic.ac.cy; 7 Biomarkers and Nutrimetabolomic Laboratory, Department of Nutrition, Food Sciences and Gastronomy, Campus Torribera, Faculty of Pharmacy and Food Sciences, University of Barcelona, CIBER de Fragilidad y Envejecimiento Saludable (CIBERFES), Instituto de Salud Carlos III, 08028 Barcelona, Spain; candres@ub.edu (C.A.-L.); margarcia@ub.edu (M.G.-A.); 8 INRA, UMR 1019, UNH, CRNH Auvergne, F-63000 Clermont-Ferrand, Clermont Université, Université d’Auvergne, Unité de Nutrition Humaine, BP 10448, F-63000 Clermont-Ferrand, France; dragan.milenkovic@clermont.inra.fr; 9 University College Dublin, D4 Dublin, Ireland; eileen.gibney@ucd.ie; 10 Université Lille, INSERM, Institut Pasteur de Lille, U1167—RID-AGE—Facteurs de Risque et Déterminants Moléculaires des Maladies Liées au Vieillissement, F-59000 Lille, France; julie.dumont@pasteur-lille.fr; 11 Department of Food and Nutritional Sciences, University of Reading, Reading RG6 6AP, UK; m.y.schar@reading.ac.uk; 12 Human Nutrition, University of Glasgow, Glasgow G31 2ER, UK; s.palma-duran.1@research.gla.ac.uk (S.A.P.-D.); Emilie.CombetAspray@glasgow.ac.uk (E.C.); 13 Goce Delcev University, 2000 Stip, Macedonia; tatjana.ruskovska@ugd.edu.mk (T.R.); viktorija.maksimova@ugd.edu.mk (V.M.); 14 Polytechnic Institute of Santarem, ESA, Department of Food Technology, Biotechnology and Nutrition, 2001-904 Santarém, Portugal

**Keywords:** flavonols, quercetin, cardiovascular disease, meta-analysis, systematic review, Interindividual variability, blood lipids, blood pressure, glucose

## Abstract

Several epidemiological studies have linked flavonols with decreased risk of cardiovascular disease (CVD). However, some heterogeneity in the individual physiological responses to the consumption of these compounds has been identified. This meta-analysis aimed to study the effect of flavonol supplementation on biomarkers of CVD risk such as, blood lipids, blood pressure and plasma glucose, as well as factors affecting their inter-individual variability. Data from 18 human randomized controlled trials were pooled and the effect was estimated using fixed or random effects meta-analysis model and reported as difference in means (DM). Variability in the response of blood lipids to supplementation with flavonols was assessed by stratifying various population subgroups: age, sex, country, and health status. Results showed significant reductions in total cholesterol (DM = −0.10 mmol/L; 95% CI: −0.20, −0.01), LDL cholesterol (DM = −0.14 mmol/L; 95% CI: −0.21, 0.07), and triacylglycerol (DM = −0.10 mmol/L; 95% CI: −0.18, 0.03), and a significant increase in HDL cholesterol (DM = 0.05 mmol/L; 95% CI: 0.02, 0.07). A significant reduction was also observed in fasting plasma glucose (DM = −0.18 mmol/L; 95% CI: −0.29, −0.08), and in blood pressure (SBP: DM = −4.84 mmHg; 95% CI: −5.64, −4.04; DBP: DM = −3.32 mmHg; 95% CI: −4.09, −2.55). Subgroup analysis showed a more pronounced effect of flavonol intake in participants from Asian countries and in participants with diagnosed disease or dyslipidemia, compared to healthy and normal baseline values. In conclusion, flavonol consumption improved biomarkers of CVD risk, however, country of origin and health status may influence the effect of flavonol intake on blood lipid levels.

## 1. Introduction

Despite the reported decline in the mortality rates of cardiovascular diseases (CVD) in the last decades, they continue to be the leading cause of morbidity and mortality in men and women in most developed countries [1,2]. Many well-recognized risk factors including hypertension, hypercholesterolemia, diabetes and obesity, as well as some lifestyle factors such as smoking, are potentially modifiable [3,4]. Thus, apart from medication, lifestyle changes and dietary modifications are crucial for preventing the incidence of CVD. Many studies have associated a high consumption of fruits and vegetables, rich in (poly)phenolic compounds, with a lower risk of chronic diseases and more specifically, CVD, although sometimes the results are inconsistent [5,6]. The mechanisms of this association are not entirely clear, but beyond nutrients, plant (poly)phenols are considered to play an important role in the maintenance of optimal cardiovascular health. Due to their effect in cell signaling pathways related to oxidative stress and inflammation, physiological conditions might be improved, including lipid metabolism, vascular function, blood pressure and glucose metabolism [7,8,9]. Despite many years of research, the evidence regarding the role of (poly)phenols in cardiovascular health is not entirely consistent. This is in part due to the heterogeneity in the design of studies, the intervention periods, and population types and size [7,10]. In addition, a high heterogeneity in individual physiological response to consumption of these bioactive compounds has been identified and has perhaps obscured associations between dietary intakes and cardioprotective effects. Factors such as the individual’s genetic background (i.e., polymorphisms), lifestyle, sex, age and gut microbiome are potential factors contributing to individual differences in the absorption, distribution, metabolism and excretion of (poly)phenols [11].

Flavonols are one of the flavonoid subclasses most common in our diet and the main food sources are vegetables such as onions, spinach, asparagus and some types of berries [12]. Within this subclass, quercetin conjugates are the most consumed type of flavonols (about 22 mg per day in Europe) [13]. Quercetin has been associated with the modulation of several cellular signaling pathways implicated in chronic diseases including cardiovascular diseases, as indicated by in vitro and animal studies [14,15]. On the other hand, epidemiological studies have significantly linked flavonol consumption with decreased risk of stroke [16,17], but not with risk of coronary heart disease [18]. Very few meta-analyses of randomized controlled trials have evaluated the effect of flavonol supplementation on cardiometabolic risk biomarkers, and, of those published, different results were obtained. Serban and collaborators have shown a significant effect in the reduction of blood pressure (mean reduction of 3.04 mm Hg in systolic blood pressure and 2.63 mm Hg in diastolic blood pressure) [19]. On the other hand, Sahebkar did not find a significant effect on triacylglycerol, LDL and HDL plasma levels, but reported a significant increase in total cholesterol (mean increase of 3.13 mg/dL [20]. Thus, to date, the clinical evidence on the beneficial effects of flavonol consumption in humans remains inconclusive, and this could be due to the interindividual variability in the response to flavonol consumption.

In the present study, we systematically reviewed all published randomized controlled trials analyzing the impact of flavonol consumption on several biomarkers of cardiometabolic risk, more specifically, blood pressure, blood lipids and glucose. The influences of factors that may be responsible for interindividual variability in the response of participants to flavonol supplementation were also investigated, including age, sex and health status.

## 2. Materials and Methods

The review was registered in PROSPERO, the international prospective register of systematic reviews (registration number: CRD42016037074).

### 2.1. Search Strategy

A systematic search was conducted in December 2015, by one of the authors in the following databases: Medline [21], SCOPUS [22], ISI Web of Knowledge [23], ClinicalTrials.gov [24] and International Clinical Trials Registry Platform [25]. Search terms in titles and abstracts included a combination with MESH terms: (1) bioactive designation and food rich in flavonol (polyphenol, flavonoid, flavonol, quercetin, kaempferol, galangin, isorhamnetin, jaceidin, kaempferide, morin, myricetin, patuletin, rhamnetin, spinacetin, rutin; spice, caper, saffron, caraway, clove, oregano, onion, shallot, broccoli, spinach, asparagus, bean, chilli pepper, berry, black chokeberry, american cranberry, and lingonberry); (2) type of study and participant (trial, experiment, study, studies, intervention; human, subjects, men, women, patient, volunteer, and participant); and (3) cardiometabolic outcome (flow mediated dilation (FMD), platelet aggregation, blood pressure (BP), total cholesterol (TC), LDL cholesterol, HDL cholesterol, triacylglycerol (TAG), body mass index (BMI), waist circumference (WC), glucose, insulin, insulin resistance (HOMA-IR), glycated hemoglobin (HbA1c) and exercise capacity). The wild-card term “(*)” was used to increase the sensitivity of the search strategy. No language restriction was used in the literature search.

Studies included in the meta-analysis were limited to human randomized controlled trials, which had a control group receiving a placebo and measured one or more of the defined outcomes (platelet aggregation and exercise capacity were considered secondary outcomes and papers with results on only these outcomes were excluded). Additional exclusion criteria were: studies with multifactorial interventions (flavonols given as a part of a multicomponent treatment; dietary or physical activity co-intervention), studies with foods having other polyphenols in higher proportions than flavonols, acute studies and studies with non-European language.

### 2.2. Data Extraction

Using a standardized data extraction form, the following data were extracted: (1) publication details—year of publication, name of first author, name and e-mail of corresponding author, and clinical trial registration number (when available); (2) sample characteristics—country, total number of participants, male and female, age mean and age range, ethnicity, health status (healthy, at risk or diagnosed disease), menopausal status, smoking, medication; baseline BMI, diet (assessment method, baseline diet and diet during study), and physical activity level; (3) study characteristics—study design (cross-over or parallel), arms number and description, washout duration, treatment duration, number of participants receiving the test bioactive or food, number of participants receiving the placebo, number of participants completing the study, composition of test and placebo, and dose and mode of administration; (4) information on reported outcomes—type of sample (fasted <8 h, 8–12 h, <12 h, and fed), equipment used (when important to outcome assessment), central measure (mean or median) and dispersion (standard deviation or standard error of the mean) with corresponding units (values before and after test, before and after placebo, or values for test and placebo changes), *p* value when available, and number of participants with adverse events. Data extraction was performed in duplicate by two authors independently and cross checked by a third author. Before analysis, outcomes on lipid levels and glucose levels were converted to mmol/L if reported in a different unit.

### 2.3. Assessment of Bias

A systematic assessment of bias in the included studies was based on the Cochrane Collaboration with some modifications [26]. The items used for the assessment of each study were as follows: (1) selection bias—random sequence generation, and allocation concealment (in each item, yes = 1; no = 0, unclear = 0); (2) performance bias—blinding (yes = 1 for each participants, researchers and statisticians, no = 0, unclear = 0), and measurement of compliance (1 for biomarker measure, 0.5 if compliance information was collected by counting non used capsules or recipients, or by self-reporting, 0 if no measurement of compliance was done or the information is insufficient); (3) attrition bias—flow of participants (1 if flow of participants is explained in detail, including number of withdrawals and reasons, 0 if there is no information or insufficient information); (4) other bias—baseline comparability between test and control groups (yes = 1, no = 0, unclear = 0), data report (1 if pre and post data or change is reported in table with central measure and spread for placebo and treatment groups, and number per group, 0 if anything is missing), and industry funding (0 if any commercial source provided some or all monetary funding for the trial, if a company carried out a study “in house”, if any of the authors was employed by a relevant industry or if it was unclear that there was any kind of industry funding, 1 if there was no funding from industry or if the only involvement of a company was to provide any ingredient for the intervention). Studies were rated as low risk of bias when total score was 8 to 10, moderate risk of bias when total score was 5 to 7.5 and high risk of bias when total score was below 5.

### 2.4. Data Analysis

Data for each outcome were analyzed using Comprehensive Meta-Analysis Software, version 3.0 (Biostat, Englewood, NJ, USA). Fixed or random effect meta-analysis were conducted to assess test/placebo differences across studies, with effect size measured as difference in means (DM) with 95% confidence intervals (random effect meta-analysis was used when high heterogeneity across studies was present). Heterogeneity of studies was assessed by the Cochran’s Q statistic (a chi-squared test with *n* − 1 degrees of freedom) and the inconsistency index *I*^2^, with a value higher than 75% being considered substantial heterogeneity [27]. Funnel plots were used to assess for evidence of publication bias. For outcomes with sufficient number of studies, the interindividual variability was assessed by exploring effects in subgroups: sex, age, country of origin and health status (BMI, healthy, with disease, and baseline values of measured outcomes and risk of cardiovascular disease). Additionally, the influence of flavonol dose, study duration and composition of test were also assessed. Sensitivity analyses were also performed by excluding studies with high risk of bias and industry funding.

Quality of evidence was assessed based on the GRADE system [28]. Level of evidence was downgraded from high to moderate in the presence of either serious risk of bias across studies or serious risk of reporting bias, and downgraded to low if both were present.

## 3. Results

### 3.1. Description of the Included Studies

**Figure 1:**
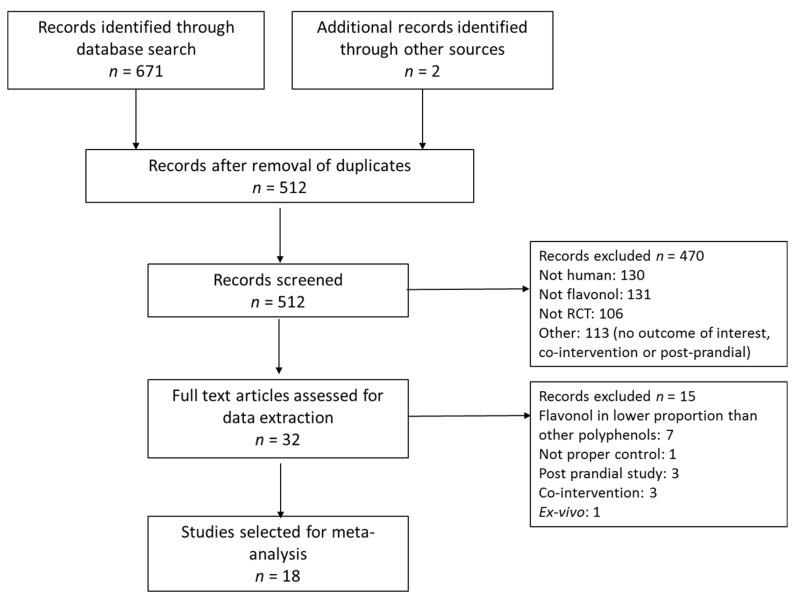
Flow diagram of study selection.

A total of 671 studies were retrieved from database search and two others from additional sources. After screening and application of exclusion criteria, 32 trials were selected for data extraction. Fifteen of these studies were rejected after detailed analysis of the full text. Many of the rejected studies in this phase were studies where the test food had a significant content in flavonols but was richer in other flavonoids or phenolic acids (for example, apple, cocoa and tea have a higher content of flavanols than flavonols; grape and berries have a higher content of anthocyanins or phenolic acids and, thus, the observed results in the outcomes cannot be imputed to the flavonols, which is the objective of this meta-analysis). Finally, 18 RCTs were included in the systematic review and meta-analysis. The study selection process is shown in Figure 1.

Characteristics of included studies are displayed in Table 1. The 18 RCTs included 530 participants from European countries: Germany [29,30,31], Ireland [32], Netherlands [33], Finland [34,35] and United Kingdom [36]; 41 participants from USA [37]; 27 participants from Canada [38]; and 388 participants from Asian countries: China [39,40], Korea [41,42,43,44] and Iran [45,46]. Nine trials followed a parallel design and nine followed a crossover design, with a duration of two to twelve weeks (Table 1). Most crossover studies were of short duration (six trials with two to four weeks of intervention) and most parallel studies had longer duration (six trials with 10 to 12 weeks of intervention) (Table 1). Interventions included pure flavonol supplements (eight trials with pure quercetin and one with myricetin) or enriched mixtures of flavonols (five extracted from onion peel, one onion juice, two extracted from sea buckthorn and one from a commercial source) (Table 1). Intervention doses ranged between 16 mg and 1200 mg of flavonol. Four trials had doses below 100 mg, seven trials had doses between 100 and 200 mg and seven trials had doses above 400mg (Table 1). These were compared to placebo versions of capsules, drinks or meals; ten of them had the same matrix as the intervention [29,32,34,35,36,37,39,41,42], five were placebo capsules containing either mannitol [31], rice flour [38], cellulose [33] or lactose [45,46] and three studies did not describe composition of placebo capsules [30,43,44]. Five of the included studies had low risk of bias, two had a high risk of bias and eleven had a moderate risk of bias (Table 1 and Supplementary Materials Table S1 for risk of bias assessment). The main measured cardiometabolic biomarkers were blood pressure (16 trials), total cholesterol, HDL and LDL (16 trials), triacylglycerol (15 trials) and glucose (10 trials). Other outcomes included BMI (seven trials), WC (four trials), insulin and HOMA-IR (four trials), HbA1c (two trials) and FMD (two trials) (Table 1).

### 3.2. Characteristics of Participants

Table 2 describes the main characteristics of the participants. From 987 subjects included in the analysis, 679 were supplemented with flavonols and 672 subjects received the placebo (325 subjects received flavonol only, 318 subjects received placebo only, and 354 received both, as enrolled in cross-over RCT). Mean age of most participants was between 40 and 50 years (eleven trials), two studies included older adults (mean ages above 59), three studies included adults with a mean age below 40 and two studies did not report mean age (Table 2). Nine studies included male and female participants, five studies included only male subjects and four studies included only female subjects (Table 2). Six studies included participants with diagnosed diseases: one with rheumatoid arthritis, one with hypertension and four with metabolic diseases—two trials with metabolic syndrome (MS), one trial with diabetes mellitus type 2 (DM2) and one trial with non-alcoholic fatty liver disease (NAFLD). The other studies described the participants as healthy subjects (thirteen trials). However, many of these studies included subjects with mild dyslipidemia (eight trials), impaired glucose tolerance (five trials), pre-hypertension or hypertension (three trials) (Table 2). Most studies had a high percentage of overweight participants (ten trials with BMI means between 25 and 29.9 kg/m^2^). The two trials with metabolic syndrome patients had a high percentage of obese subjects (BMI means above 30 kg/m^2^). Only four studies reported normal BMI means (below 25 kg/m^2^) and two studies did not report BMI values (Table 2). Smoking, an important risk factor of cardiometabolic diseases, was not reported in five studies; two studies had a mixed population of smokers and non-smokers, one study only assessed smokers and ten studies included only non-smokers (Table 2). Other lifestyle factors that may influence the effect of flavonol supplementation on the measured outcomes, such as baseline diet and physical activity level, were reported in very few trials. One trial reported energy intake at baseline [39], three reported energy and macronutrient [29,38,41,46] and one reported consumption of vegetables and fruits [35]. Only one trial reported baseline physical activity level [46]. Most of the studies had no dietary restrictions either before or during the intervention period (fourteen trials [29,30,31,34,35,36,37,38,39,40,43,44,45,46]); two trials restricted quercetin rich foods before the study [41,42], one trial restricted flavonoid rich foods during the intervention [33] and one trial restricted the intake of wine during the intervention [32].

### 3.3. Effect of Flavonol Supplementation on Biomarkers of Cardiometabolic Risk

#### 3.3.1. Blood Lipids

**Figure 2:**
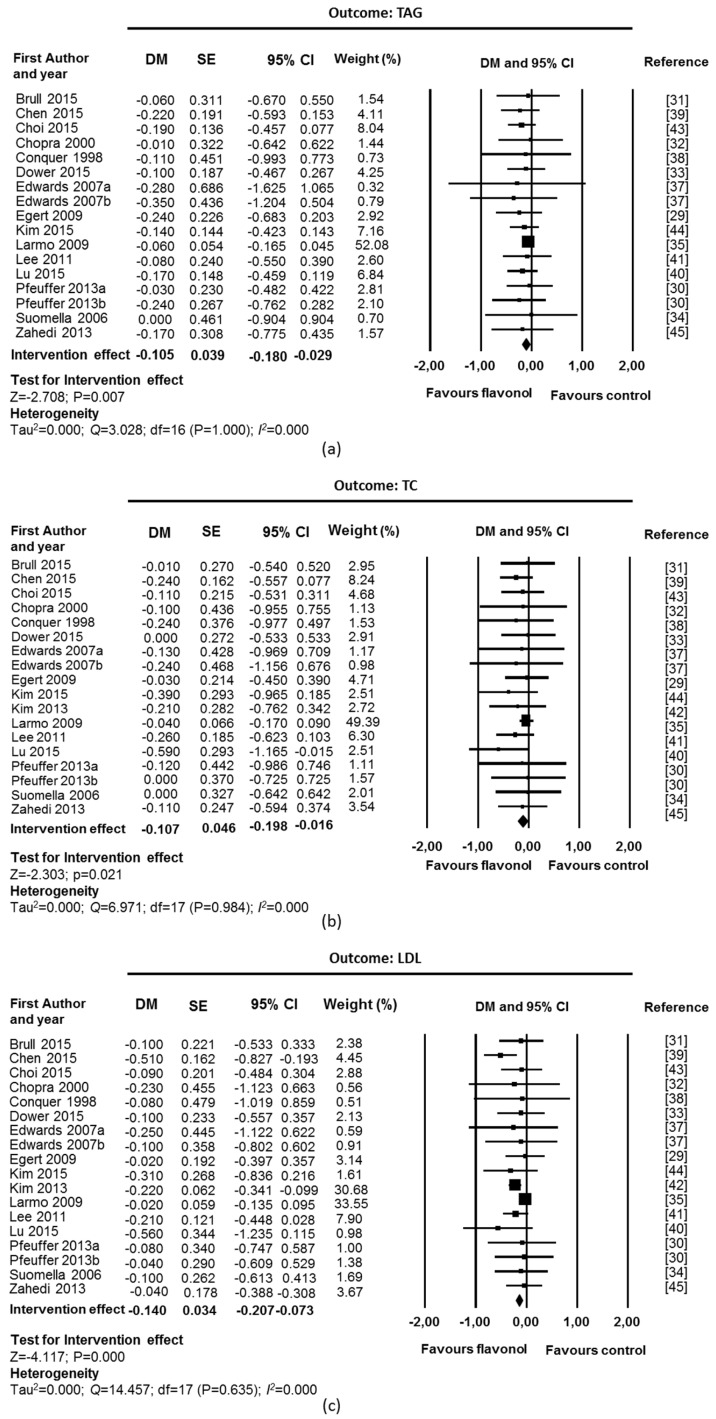

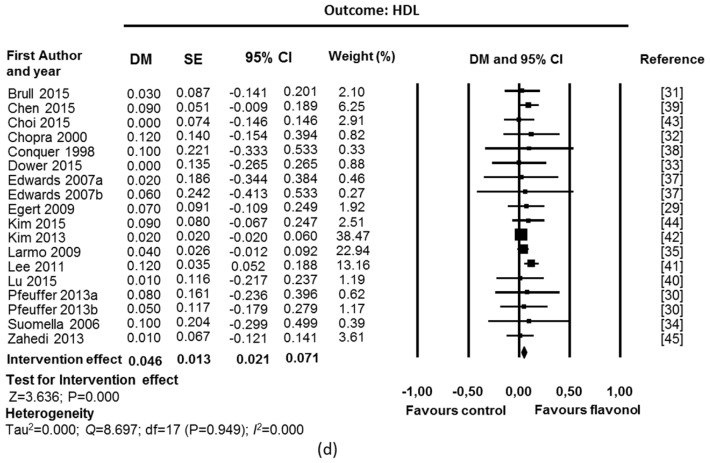
Effect of flavonol supplementation on measures of blood lipids (mmol/L): (**a**) Triacylglycerols (TAG); (**b**) Total Cholesterol (TC); (**c**) LDL Cholesterol (LDL); and (**d**) HDL Cholesterol (HDL). All studies were used for fixed effect model meta-analysis. The Edwards study consisted of two substudies: hypertensive participants (Edwards 2007a) and pre-hypertensive participants (Edwards 2007b). The Peuffer study also consisted of two substudies: polymorphism ApoE3 (Pfeuffer 2013a) and ApoE4 (Pfeuffer 2013a). DM: difference in means, SE: standard error, CI: confidence interval.

Chronic supplementation with flavonols, with quercetin as the main flavonol used in the interventions, was associated with a beneficial effect on blood lipid levels (Figure 2). Fixed-effect meta-analyses showed a significant reduction in TAGs levels (DM = −0.10 mmol/L; CI: −0.18, −0.03; *p* = 0.007; 17 studies, 467 supplemented participants and 456 control participants, Figure 2a), TC (DM = −0.11 mmol/L; CI: −0.20, −0.02; *p* = 0.021; 18 studies, 473 supplemented participants and 462 control participants; Figure 2b) and LDL (DM = −0.14 mmol/L; CI: −0.21, −0.07; *p* = 0.000; 18 studies, 473 supplemented participants and 462 control participants; Figure 2c). A significant increase was observed in HDL (DM = 0.05 mmol/L; CI: 0.02, 0.07; *p* = 0.000; 18 studies, 473 supplemented participants and 462 control participants; Figure 2d). There was no suggestion of heterogeneity among studies (*I*^2^ = 0% and *p* value for Q test > 0.1). Funnel plots and Egger’s weighted regression statistic did not suggest evidence of publication bias for TC (*p* = 0.089), HDL (*p* = 0.461), and LDL (*p* = 0.494). However, asymmetry was detected for TAG (*p* = 0.048) (Supplementary Materials Figure S1).

Sensitivity analyses were performed removing studies with high risk of bias [32,41] and industry funding [33]. In LDL and HDL, a small study (12 participants), which had a high relative weight due to a very small variance compared to the other studies was also removed [42]. The intervention effect remained significant for all outcomes (TAG: DM = −0.11 mmol/L; CI: −0.19, −0.03; *p* = 0.007; 14 studies, 390 supplemented participants and 386 control participants; TC: DM = −0.10 mmol/L; CI: −0.20, −0.00; *p* = 0.042; 15 studies, 386 supplemented participants and 392 control participants; LDL: DM = −0.09 mmol/L; CI: −0.18, −0.00; *p* = 0.043; 14 studies, 390 supplemented participants and 386 control participants; HDL: DM = 0.05 mmol/L; CI: 0.01, 0.08; *p* = 0.013; 14 studies, 390 supplemented participants and 386 control participants).

#### 3.3.2. Blood Pressure

**Figure 3:**
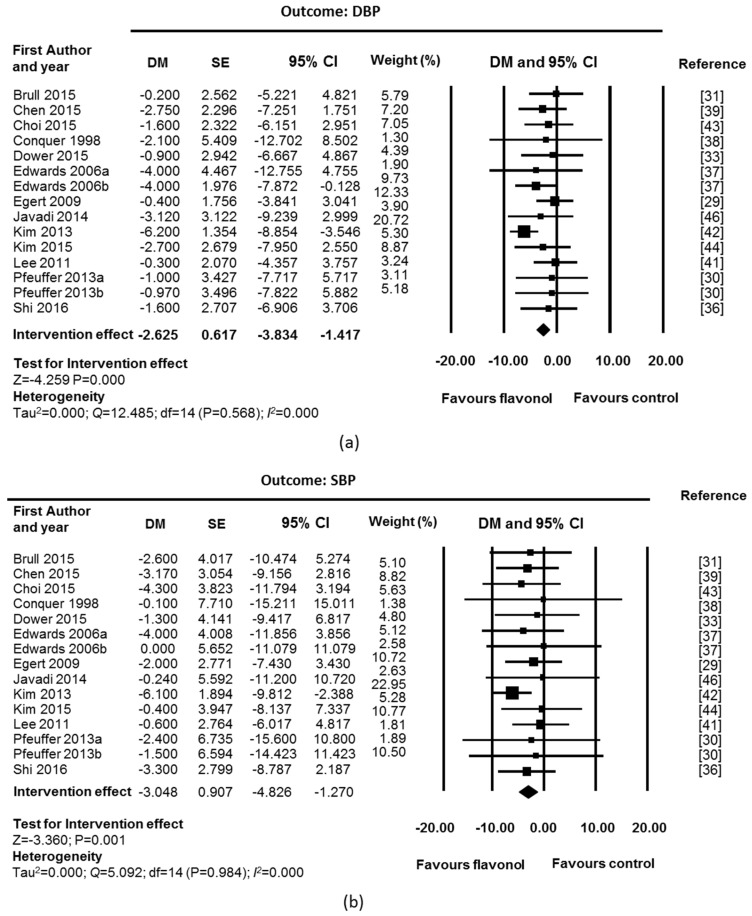
Effect of flavonol supplementation on measures of blood pressure (mmHg): (**a**) Dyastolic blood pressure (DBP); and (**b**) Systolic blood pressure (SBP). All studies were used for fixed effect model meta-analysis. The Edwards study consisted of two substudies: hypertensive participants (Edwards 2007a) and pre-hypertensive participants (Edwards 2007b). The Pfeuffer study also consisted of two substudies: polymorphism ApoE3 (Pfeuffer 2013a) and ApoE4 (Pfeuffer 2013b).

A beneficial effect in blood pressure was also observed after chronic supplementation with flavonols (Figure 3). Fixed-effect meta-analyses showed a significant reduction for DBP and SBP (DBP: DM = −2.62 mmHg; CI: −3.83, −1.42; *p* = 0.000; SBP: DM= −3.05 mmHg; CI: −4.83, −1.27; *p* = 0.001; 15 studies, 336 supplemented participants and 334 control participants). No heterogeneity was found in any of the outcomes (*I*^2^ = 0% and *p* value for Q test > 0.1). Analysis of funnel plots and Egger’s statistics (Supplementary Materials Figure S1) suggested the existence of asymmetry for SBP (*p* = 0.018), suggesting the existence of publication bias.

In DBP and SBP, sensitivity analysis was performed by removing the study with high risk of bias [41] and the industry funded study [33].The intervention effect was maintained after removal (DBP: DM = −2.95 mmHg; CI: −4.25, −1.65, *p* = 0.000; SBP: DM = −3.46 mmHg; CI: −5.39, −1.52, *p* = 0.000; 13 studies, 269 supplemented participants, 274 control participants).

#### 3.3.3. Fasting Glucose

**Figure 4:**
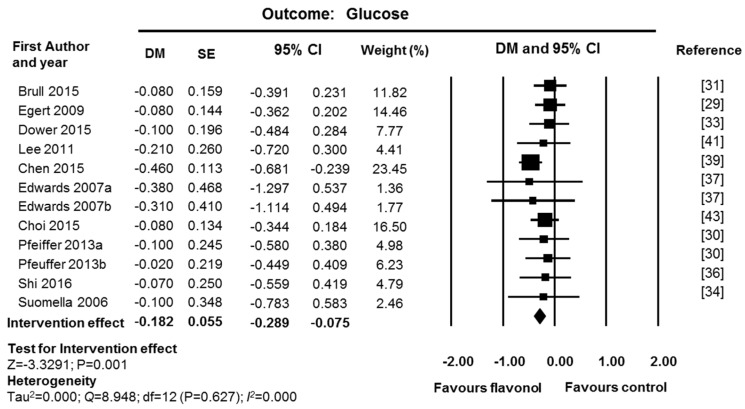
Effect of flavonol supplementation on measures of plasma glucose (mmol/L). All studies were used for fixed effect model meta-analysis. The Edwards study consisted of two substudies: hypertensive participants (Edwards 2007a) and pre-hypertensive participants (Edwards 2007b). The Pfeuffer study also consisted of two substudies: polymorphism ApoE3 (Pfeuffer 2013a) and ApoE4 (Pfeuffer 2013b).

Fixed-effect meta-analysis of glucose data showed a significant reduction in fasting glucose levels after supplementation with flavonols (DM = −0.18 mmol/L; CI: −0.29, −0.08; *p* = 0.001; 12 studies, 276 supplemented participants and 270 control participants; Figure 4). No heterogeneity was found (*I*^2^ = 0% and *p* value for Q test > 0.1). Sensitivity analysis was performed by removing one study with high risk of bias [41] and one industry funded study [33]. The intervention effect and the significance level were maintained (DM = −0.19 mmol/L; CI: −0.30, −0.07, *p* = 0.001; 10 studies, 209 supplemented participants and 205 control participants). Analysis of funnel plots and Egger’s statistics (Supplementary Materials Figure S1) showed no evidence of publication bias for glucose (*p* = 0.441).

### 3.4. Subgroup Analyses for Identification of Factors Affecting Inter-Individual Variability

To explore the factors that could influence the individual response to flavonol intake on the studied outcomes, subgroup analysis was performed on blood lipids. Studies with high risk of bias were excluded from the subgroup analysis [32,41]. As in the sensitivity analysis (Section 3.3.1), another study was also excluded in LDL and HDL subgroup analyses [42]. Subgroup analysis on blood pressure was not performed due to a lower number of studies and evidence of publication bias. Subgroup analysis on fasting glucose was also not undertaken due to the low number of studies available. Table 3 presents a summary of the results obtained for subgroup analysis performed on participant characteristics such as age, sex, country of origin, and health status (BMI, diagnosed disease, baseline lipid levels and cardiovascular risk) (the complete data of the subgroup analyses are presented on Supplementary Materials Table S2).

#### 3.4.1. Stratification by Age, Sex, and Country

Based on participants age range, two subgroups of age were defined: eleven studies were categorized as “mixed ages” (700 participants with ages from 19 to 70 years) [29,31,34,35,37,39,40,43,44,45], and four as “age above 40 years” (111 participants, ages from 40 to 79 years) [30,33,38]. Only one study included younger adults, with ages below 25 (12 participants) [42]. A significant intervention effect was found in the mixed ages subgroup for TAG, LDL, and HDL, which was lost in the older age group, possibly due to the lower sample size (Table 3). No heterogeneity was observed between subgroups of mixed ages and ages above 40 years (Supplementary Materials Table S2).

Most studies included both sexes but did not differentiate results for men and women (mixed sex subgroup, 10 studies, 649 participants) [29,31,33,35,37,38,39,40,43]. Comparison of studies performed with only men or women (two studies with 99 females [42,44,45]; three studies with 63 males [30,34]) was inconclusive due to the low number of subjects in either subgroup (Table 3).

Ethnicity of participants was poorly described in the selected studies, and country of recruitment was used for stratification in two subgroups: Asian countries (China [39,40], Korea [43,44] and Iran [45]; five studies, 255 participants) and countries from Europe and North America (Europe [29,30,31,33,34,35], USA [37] and Canada [38]; 10 studies, 556 participants). Reductions of TAG, TC and LDL after intervention are more pronounced and significant in studies undertaken in Asia (Table 3). The effect of flavonol intake on HDL was not observed when considering the subgroups only (Table 3). Evidence of heterogeneity between the two subgroups was found for LDL (*p* value for Q test = 0.024), emphasizing the potential role of the participants’ characteristics in the observed difference in the intervention effect (Supplementary Materials Table S2).

#### 3.4.2. Stratification by Health Status

Most of the studies included participants with BMI values from normal weight to overweight (mixed BMI subgroup, eight studies, 246 participants) [30,33,34,38,39,40,44], showing significant effects of flavonol intake on all blood lipids (Table 3). A significant effect was not observed for TC, LDL and HDL, in the overweight subgroup (five studies, 274 participants) [29,31,37,43] and in the normal weight subgroup [35] (one study, 229, low risk of bias) (Table 3). A more pronounced effect of flavonol intake was observed for TAG in the overweight subgroup (Table 3), with a *p*-value near the significance cutoff (Supplementary Materials Table S2). No heterogeneity was found between BMI subgroups in all outcomes, except for LDL (Supplementary Materials Table S2).

Regarding disease status, participants were divided in two subgroups: participants with no diagnosed disease (eight studies, 399 participants) [30,34,35,37,38,40,44] and participants with diagnosed cardiometabolic diseases (five studies, 305 participants) [29,31,37,39,45]. Two studies with mixed health status, including both healthy and hypertensive participants, were excluded from this subgroup analysis [33,43]. Results suggest a tendency for a more pronounced intervention effect in the disease subgroup than in the no disease subgroup (Table 3). However, a significant intervention effect was only observed in the disease subgroup for LDL (Table 3). There was no suggestion of heterogeneity between subgroups (Supplementary Materials Table S2).

When studies were stratified according to baseline lipid levels, two subgroups were defined: normal and dyslipidemic (see Supplementary Materials Table S2 for data). Studies that included both normal and dyslipidemic participants were excluded (HDL: [33]; LDL: [43], TC: [37,39]). A significant impact of flavonol intake was only detected for TAG and HDL, in the dyslipidemic subgroup (Table 3). No significant intervention effect was observed in the normal baseline subgroup. No evidence of heterogeneity across subgroups was observed (Supplementary Materials Table S2).

### 3.5. Influence of the Type and Dose of Flavonol

Studies were stratified according to the type and dose of flavonol used in the intervention. Studies with high risk of bias were removed. Regarding the type of flavonol (pure or mixture), a more pronounced intervention effect was observed on TAG and LDL only (Table 4), after administration of a pure compound, compared to a mixture of flavonols. When a study with pure myricetin was removed from the subgroup of pure compounds [39] (all other studies used quercetin as the pure flavonol), the intervention effect with pure flavonol was no longer significant on LDL (pure subgroup: DM = −0.06, CI: −0.25, 0.12; *p* = 0.514; mixture subgroup: DM = −0.06; CI: −0.16, 0.04; *p* = 0.281). On TAG, the effect was reversed, with the mixture subgroup showing a significant intervention effect and the pure subgroup a non-significant intervention effect (pure subgroup: DM = −0.16; CI: −0.36, 0.04; *p* = 0.114; mixture subgroup: DM = −0.09; CI: −0.18, −0.00; *p* = 0.039).

Studies were stratified in two dose groups: (1) below 200 mg of flavonol per day (low dose studies); and (2) above 200 mg per day (high dose studies). No significant effect was observed for TC in either subgroup, with an effect remaining for TAG and HDL in the low dose studies, and for LDL in high dose studies (Table 4). No evidence of heterogeneity between subgroups was observed (*p* value for Q test > 0.1, *I*^2^ = 0%) for all blood lipids, except for LDL (*p* value for Q test = 0.072, *I*^2^ = 6.21%).

## 4. Discussion

### 4.1. Quality of Evidence and Clinical Importance of the Observed Effects

The present meta-analysis of RCTs assesses the effect of flavonol supplementation on several cardiometabolic risk factors. Results showed that medium-term flavonol intake exerted a beneficial effect on blood pressure, blood lipids profile, and fasting glucose levels (Table 5). A previously published meta-analysis of RCTs assessing the effects of quercetin on blood pressure, Serban et al., reported a BP-lowering effect of 3.04 mmHg for SBP and 2.63 mmHg for DBP [19]. The present meta-analysis pooled data from the same studies as Serban et al., and five more studies published in 2015, showing a higher lowering effect (Table 5). As shown for quercetin, the possible mechanisms behind the observed blood pressure decrease may be associated with an improvement of endothelial function by increasing NO (nitric oxide) production and/or bioavailability, by modulation of oxidative stress, and by interference with the renin-angiotensin-aldosterone system (RAAS). Direct renal effects might also play some role in the antihypertensive effect, such as the downregulation of epithelial Na^+^ channel (ENaC) in the kidney [50,51,52].

The present meta-analysis also showed a beneficial effect on blood lipids profile (Table 5) contrary to the one published by Sahebkar [20], who reported no effect on TAG, LDL and HDL, and a small increase in TC (0.08 mmol/L). This may result from the fact that Sahebkar only assessed RCTs with pure quercetin aglycone and quercetin dehydrate. The present meta-analysis pooled results from the six studies used by Sahebkar and ten more studies that also included mixtures of flavonols. To our knowledge, this is the first meta-analysis showing a lowering effect of flavonol intake on fasting glucose levels.

Previously published meta-analyses of RCTs have evaluated the effect of other (poly)phenols, or foods rich in other (poly)phenols, on cardiometabolic risk factors. Similar lowering effects on BP were reported with pomegranate juice (−4.96/−2.01 mmHg, SBP/DBP) [53]. On the other hand, grape (poly)phenols, green tea and cocoa had smaller effects on blood pressure than the present meta-analysis (−1.54/non-significant mmHg for grape seed extract [54]; −1.98/−1.92 mm Hg for green tea [55]; and non-significant/−1.60 mmHg for cocoa [56], SBP/DBP), and anthocyanins had no significant effects on blood pressure [57]. On blood lipids, no significant effects were reported for pomegranate juice and grape seed extract meta-analyses [54,58]. Meta-analyses of soy products [59] and black tea [60] reported lowering effects on LDL (−0.12 mmol/L and −0.14 mmol/L), similar to the present meta-analysis, but smaller or non-significant effects in the other lipid levels. Cocoa products and flavan-3-ols also seem to have a smaller effect on lipid levels than the reported for flavonols in the present meta-analysis (non-significant for TAG and TC, −0.07 mmol/L for LDL and 0.03 mmol/L for HDL), and no significant effect on fasting glucose levels [56].

Most of the randomized controlled trials presented here had a moderate to low risk of bias (Supplementary Materials Table S1). Based on the GRADE system [28] the quality of evidence for the intervention effect was evaluated as moderate for TC, LDL, HDL and glucose and low for TAG and blood pressure (Table 5). No high-quality evidence was achieved due to many studies with unclear reporting of allocation concealment (60% for glucose, 69% for blood lipids and 71% for blood pressure), unclear or no blinding of researchers (30% for glucose, 43% for blood pressure and 44% for blood lipids), incomplete description of participants’ flow (28% for blood pressure, 30% for glucose and 44% for blood lipids) and serious evidence of reporting bias for TAG and blood pressure (Table 5). Strengths included the absence of heterogeneity across studies and the fact that the observed intervention effect was stable to sensitivity analysis in all the analyzed biomarkers.

The observed intervention effects have clinical relevance in patients with stage 1 hypertension, mild dyslipidemia, and impaired fasting glucose. For example, for blood pressure, the observed mean reduction (4.84/3.32 mmHg, SBP/DBP) would lower stage 1 hypertensive values (140/90 mmHg, SBP/DBP), to pre-hypertensive values (135.2/86.7 mmHg, SBP/DBP) [47], corresponding to a mean reduction of 3% to 4% (SBP/DBP). Furthermore, it has been estimated that a reduction of blood pressure by 2 to 5 mm Hg may reduce risk of stroke by 6% to 14% and risk of coronary heart disease by 4% to 9% [61]. For glucose levels, the observed lowering effect of 0.18 mmol/L would lower values of mild impaired fasting glucose to normal values (below 5.6 mmol/L [49]), corresponding to a mean reduction of 3%. The lowering effect observed for blood lipids is less pronounced (0.1 mmol/L for TAG and TC, 0.14 mmol/L for LDL and 0.05 for HDL). Nevertheless, it may lower mild dyslipidemic values to normal values (below 1.7/5.2/2.6 mmol/L for TAG/TC/LDL [48]), representing a mean reduction of 6% for TAG, 5% for LDL and 2% for TC. In patients with mild dyslipidemia, it has been reported that different approaches, such as introduction of non-drug lowering substances to a healthy diet, physical activity and weight management, should be expected to reduce LDL by 3% to 7% each, and when combined, reach a lowering effect of about 10% to 20% [62]. Thus, supplementation with flavonols, may be used as another potential adjunct to lower global risk of cardiovascular diseases by controlling blood lipid levels, as well as blood pressure and glucose levels, either in single or multiple risk factors patients.

### 4.2. Inter-Individual Variability

To study the inter-individual variability in response to flavonol supplementation, the present meta-analysis stratified the participants in different subgroups of age, sex, BMI, disease status and baseline blood lipid levels. However, the low number of studies was a serious limitation to the power of meta-analysis in the subgroups, particularly in age, sex and BMI subgroups, leading to inconclusive results. A higher number of studies would allow for robust meta-regression analyses, which could give more insight into the impact of inter-individual variability.

On the other hand, meta-analysis results obtained after stratification by country, disease status and baseline lipid levels have shown for the first-time evidence that ethnicity and health status may influence the response of participants to flavonol supplementation. Participants from Asian countries showed higher reductions in TAG, TC and LDL, and higher increases in HDL after flavonol supplementation, compared to European and North American participants. The influence of health status on the results obtained for country subgroup analysis were tested by performing the analysis after removal of participants with diagnosed diseases and stratification of remaining studies between Asian and Europe and North American countries. Higher and significant reductions were maintained for Asian participants on TAG and TC, compared to the subgroup of Europe and North America (Supplementary Materials Table S3), suggesting that flavonol supplementation may be more effective in lowering TAG and TC values in Asian individuals, than in individuals from Europe and North American countries.

Regarding health status, significant intervention effects were observed for LDL in participants with diagnosed diseases, for HDL in healthy individuals, and for TAG, and HDL in participants with dyslipidemia. To remove the potential influence of ethnicity, analyses of disease status subgroups and baseline levels subgroups were repeated only with EU and North America countries. Significant intervention effects were maintained only for LDL in the disease subgroup and HDL in the dyslipidemia subgroup; no significant effects were observed in healthy participants, or participants with normal baseline levels for any of the blood lipids (Supplementary Materials Table S3). A meta-analysis on the effect of cocoa consumption on blood lipids, has also observed a significant reduction in TC and LDL in participants with cardiovascular risk, but no effect on healthy participants [63]. Similarly, a meta-analysis on the effect of soy products has reported a higher lowering effect for LDL on hypercholesterolemic participants [59]. On the contrary, results from a meta-analysis on the effect of black tea suggest a significant lowering effect for LDL on healthy participants, but not on patients with coronary artery disease [60].

This meta-analysis has several strengths and limitations. Strengths included the absence of heterogeneity in effect size for all the biomarkers analyzed, the direction of the intervention effect was stable to sensitivity analysis and there was a low number of studies with high risk of bias. Another strength is that all RCTs used a pure or enriched mixture of flavonols, instead of food, which has a more complex matrix, with other phytochemicals and nutrients that may also influence the intervention effect. One limitation was the low number of studies, which hindered the definition of well-defined subgroups for analysis, particularly age, sex, and BMI subgroups. Another limitation was the lack of information on some lifestyle habits in most of the published RCTs, which are known to influence cardiometabolic biomarkers [3], namely, out of the 18 trials, 6 did not report smoking habits, 11 did not report baseline diet, and 17 did not report physical activity level. Another important aspect, particularly in lipid profiles and cardiovascular risk assessment, is apolipoprotein E (APOE) genotype, with APOE4 having a more atherogenic profile than APOE3 and thus being considered as non-traditional CVD risk [48]. Only two studies have differentiated between participants with APOE3 and APOE4 genotype [29,30], but results from these trials had different results and were inconclusive regarding the effect of the APOE genotype on the response to flavonol supplementation. Finally, no conclusions on the real effect of long term supplementation with flavonol can be drawn, because only one study was conducted for three months [30] and many studies were short-term [32,33,34,36,37,38,42]. Two meta-analyses of observational large cohort studies have shown that a long term high intake of flavonols, compared with a low intake was inversely associated with nonfatal and fatal stroke [16]. Moreover, an increase of 20 mg of flavonol intake was associated with a 14% decrease risk for developing stroke [17].

## 5. Conclusions

The co-existence of risk factors such as dyslipidemia, hypertension and diabetes adds to the risk of cardiovascular disease. The results of this meta-analysis suggest that intake of flavonols, particularly quercetin, has a beneficial effect on blood lipids, glucose and blood pressure, contributing to lower global risk of cardiovascular disease. In the future, large-scale studies with direct clinical endpoints should be conducted to further corroborate the findings of this meta-analysis and establish a causal link between a diet rich in flavonols and cardiovascular health. Our data also show, for the first time, that some individual characteristics may influence this beneficial effect, particularly, ethnicity, disease, and baseline levels. This is an important subject for further research if we want to translate the observed responses to clinical practice and adequate diet and lifestyle recommendations for different populations: men and women, young and older adults, healthy, diseased or at risk. More RCTs need to be performed, assuring a low risk of bias and reporting all the characteristics that may influence the response, preferably differentiated for men and women: mean and range of age and BMI, presence of genetic polymorphisms, number and percentage of different ethnicities, health status (including disease, medication, baseline levels of cardiometabolic markers) and lifestyle factors such as smoking habits, baseline diet and baseline physical activity level. Finally, to allow a pooled analysis of a high number of studies and a higher power of systematic reviews and meta-analysis, a database of pre and post data for each analyzed outcome should be created and made available for the scientific community.

## Figures and Tables

**Table 1:** Characteristics of selected RCTs examining the effect of flavonol supplementation on cardiometabolic biomarkers.

First Author, Year Country (Reference)	Type of RCT N_T_/N_C_ Duration	Dose of Flavonol/Day ^1^	Cardiometabolic Markers ^2^	Risk of Bias ^3^
Brull, 2015 Germany [31]	Crossover 68/68 42 days	162 mg quercetin (OPE)	BP, TC, HDL, LDL, TAG, Glucose, Insulin, HOMA-IR, HbA1c	Low
Chen, 2015 China [39]	Parallel 30/30 84 days	600 mg dihydromyricetin (commercial)	BMI, BP, TC, HDL, LDL, TAG, Glucose, Insulin, HOMA-IR	Low
Choi, 2015 Korea [43]	Parallel 34/38 84 days	100 mg quercetin (OPE)	FMD, BMI, BP, TC, HDL, LDL, TAG, Glucose	Moderate
Chopra, 2000 Ireland [32]	Crossover 20/20 14 days	30 mg quercetin aglycone (commercial)	TC, HDL, LDL, TAG	High
Conquer, 1998 Canada [38]	Parallel 13/14 28 days	1000 mg quercetin + 200 mg rutin (commercial)	BP, TC, HDL, LDL, TAG	Moderate
Dower, 2015 Netherland [33]	Crossover 35/35 28 days	160 mg quercetin-3-glucoside	FMD, BP, TC, HDL, LDL, TAG, Glucose, Insulin, HOMA-IR	Low
Edwards, 2007 USA [37]	Crossover 41/41 28 days	728 mg quercetin aglycone (commercial)	BMI, BP, TC, HDL, LDL, TAG, Glucose	Moderate
Egert, 2009 Germany [29]	Crossover 93/93 42 days	150 mg quercetin dehydrate (commercial)	BP, TC, HDL, LDL, TAG, Glucose	Low
Javadi, 2014 Iran [46]	Parallel 20/20 56 days	500 mg quercetin (commercial)	BP	Moderate
Kim, 2013 Korea [42]	Crossover 12/12 14 days	100 mg quercetin (OPE)	BP, TC, HDL, LDL	Moderate
Kim, 2015 Korea [44]	Parallel 18/19 84 days	100 mg quercetin (OPE)	BMI, WC, BP, TC, HDL, LDL, TAG	Moderate
Larmo, 2009 Finland [35]	Parallel 115/114 90 days	13 mg isorhamnetin + 3 mg quercetin (SBP)	TC, HDL, LDL, TAG	Low
Lee, 2011 Korea [41]	Parallel 49/43 70 days	400 mg quercetin (OPE)	BMI, WC, BP, TC, HDL, LDL, TAG, Glucose	High
Lu, 2015 China [40]	Parallel 12/12 56 days	6 mg quercetin (onion juice)	BMI, WC, TC, HDL, LDL, TAG	Moderate
Pfeuffer, 2013 Germany [30]	Crossover 49/49 56 days	150 mg quercetin dehydrate (commercial)	BMI, WC, BP, TC, HDL, LDL, TAG, Glucose, Insulin, HOMA-IR, HbA1c	Moderate
Shi, 2016 UK [36]	Crossover 22/22 28 days	500 mg quercetin aglycone (commercial)	BP, Glucose	Low
Suomela, 2006 Finland [34]	Crossover 14/14 28 days	54 mg isorhamnetin + 20 mg quercetin (SBE)	TC, HDL, LDL, TAG, Glucose	Moderate
Zahedi, 2013 Iran [45]	Parallel 34/28 70 days	500 mg quercetin (commercial)	TC, HDL, LDL, TAG	Moderate

^1^ OPE—onion peel extract; SBP—sea buckthorn puree; SBE—sea buckthorn extract; ^2^ BP—blood pressure, BMI—body mass index, WC—waist circumference, TC—total cholesterol, LDL—low density lipoprotein, HDL—high density lipoprotein, TAG—Triacylglycerol; ^3^ See Supplementary Materials Table S1 for risk assessment, N_T_—number of participants supplemented with flavonol; N_C_—number of participants receiving placebo.

**Table 2:** Characteristics of participants.

First Author, Year (Reference)	Age (Mean, SD or Range) Sex (*n*M/*n*F)	Health Status ^1^ BMI (Mean, SD) Healthy/Disease Med/Smoking	Fasting Baseline Values ^2^
Brull, 2015 [31]	47.4 (10.5) 34 M/34 F	31.1 (3.4) MS Med/non smokers	BP: Pre-HT/HT TC, HDL, LDL, TAG: borderline Glucose: normal
Chen, 2015 [39]	45.1 (10.0) 40 M/20 F	25.6 (2.6) NAFLD Not Med/smoking NR	BP: normal/pre-HT TC: normal/borderline, HDL: borderline LDL: normal, TAG: high Glucose: normal/IFG
Choi, 2015 [43]	43.1 (6.7) 11 M/51 F	26.6 (2.9) Healthy Med NR/5% smokers	BP: normal/pre-HT TC: normal,HDL: borderline LDL: normal/borderline, TAG: normal, Glucose: NR
Chopra, 2000 [32]	46 (SD NR) 20 M	BMI NR Healthy Med NR/non smokers	BP: NR, TC, HDL, LDL: borderline, TAG: normal; Glucose: NR
Conquer, 1998 [38]	42.0 (13.5) M/F (*n* NR)	26.1 (4.4) Healthy Med NR/smoking NR	BP: normal TC, HDL, LDL,TAG: normal; Glucose: NR
Dower, 2015 [33]	66.4 (7.9) 25 M/12 F	26.7 (3.3) Healthy Not Med/non smokers	BP: normal/pre-HT TC, LDL: borderline, HDL: normal/borderline, TAG: normal Glucose: normal/IFG
Edwards, 2007 [37] Pre-HT group	47.8 (15.2) 13 M/6 F	29.8 (5.7) Healthy Not Med/non smokers	BP: pre-HT/HT1 TC, LDL: normal/borderline HDL: borderline, TAG—borderline/high Glucose: normal/IFG
HT1 group	49.2 (13.6) 13 M/9 F	29.5 (6.6) HT Not Med/non smokers	
Egert, 2009 [29]	45.1 (10.5) 42 M/51 F	30.6 (3.2) MS Med/non smokers	BP: normal/pre-HT TC, HDL, LDL, TAG: borderline/high Glucose: normal/IFG
Javadi, 2014 [46]	47.3 (9.1) 40 F	29.3 (4.5) RA Med/non smokers	BP: normal TC, HDL, LDL, TAG: NR Glucose: NR
Kim, 2013 [42]	20–25 12 F	20.2 (1.7) Healthy Not Med/smoking NR	BP: normal TC: NR HDL, LDL,TAG: normal Glucose: NR
Kim, 2015 [44]	45.0 (8.5) 37 F	26.6 (3.2) Healthy Not Med/smoking NR	BP: normal TC, LDL, TAG: normal HDL: borderline Glucose: NR
Larmo, 2009 [35]	30.8 (8.7) 51 M/178 F	23.1 (2.9) Healthy Not Med/7% smokers	BP: NR TC, HDL, LDL, TAG: normal Glucose: NR
Lee, 2011 [41]	44.2 (7.8) 92 M	24.8 (2.9) Healthy Not Med/smokers	BP: normal/pre-HT TC, LDL: normal HDL,TAG: borderline Glucose: normal/IFG
Lu, 2015 [40]	35–55 10 M/13 F	25.5 (2.6) Healthy Not Med/non smokers	BP: NR TC, HDL, LDL: borderline TAG: normal Glucose: NR
Pfeuffer, 2013 [30]	59.4 (6.3) 49 M	26.3 (2.1) Healthy Not Med/non smokers	BP: pre-HT TC, HDL, LDL: borderline TAG: normal Glucose: normal/IFG
Shi, 2016 [36]	29.9 (12.9) 22 M	24.8 (3.0) Healthy Med NR/non smokers	BP: normal/pre-HT TC, HDL, LDL, TAG: NR Glucose: normal/IFG
Suomela, 2006 [34]	46.6 (5.6) 14 M	25.8 (SD NR) Healthy Not Med/non smokers	BP: NR TC, HDL, LDL: borderline TAG: normal Glucose: normal
Zahedi, 2013 [45]	35–55 62 F	BMI NR DM 2 Med/non smokers	BP: normal TC, LDL: normal HDL, TAG: borderline Glucose: NR

^1^ MS—Metabolic Syndrome; NAFLD—Non Alcoholic Fatty Liver Disease; HT—Hypertension, RA—Reumathoid Arthritis; DM 2—Diabetes Mellitus type 2; ^2^ Blood pressure (BP) levels (mmHg)—Normal <120 systolic/<80 diastolic; Pre-hypertension (Pre-HT) 120–139 systolic/80–89 diastolic; Hypertension stage 1 (HT1) 140–159 systolic/90–99 diastolic [47]. Fasting lipid levels (mmol/L)—Normal: Total Cholesterol (TC) < 5.2, LDL < 2.6, HDL ≥ 1.5, Triacylglycerol (TAG) < 1.7; Borderline: TC 5.2–6.2, LDL 3.4–4.1, HDL 1–1.5 (M), 1.3–1.5 (F), TAG 1.7–2.2; High risk: (TC) ≥ 6.2, LDL ≥ 4.1, HDL < 1.0 (M), < 1.3 (F), TAG ≥ 2.3 [48]. Fasting plasma glucose levels (mmol/L)—normal <5.6; Impaired fasting glucose (IFG) 5.6–6.9; diabetes ≥ 7.0 [49]. M—male; F—female.

**Table 3:** Subgroup analysis on blood lipid biomarkers: stratification by participants’ characteristics.

Factor Subgroups	TAG (mmol/L) DM (95% CI) ^1^	TC (mmol/L) DM (95% CI) ^1^	LDL (mmol/L) DM (95% CI) ^1^	HDL (mmol/L) DM (95% CI) ^1^
**Age**				
≥40	−0.11 (−0.35, 0.13)	−0.03 (−0.41, 0.36)	−0.08 (−0.38, 0.22)	0.05 (−0.10, 0.19)
Mixed	−0.11 (−0.19, −0.02) *	−0.10 (−0.19, −0.00)	−0.09 (−0.18, −0.00) *	0.04 (0.01, 0.08) *
**Sex**				
F	−0.14 (−0.40, 0.11)	−0.22 (−0.53, 0.09)	−0.12 (−0.41, −0.17)	0.04 (−0.06, 0.14)
M	−0.10 (−0.42, 0.22)	−0.03 (−0.45, 0.39)	−0.07 (−0.41, 0.26)	0.07 (−0.10, 0.23)
Mixed	−0.10 (−0.19, −0.02) *	−0.08 (−0.19, 0.02)	−0.09 (−0.18, 0.01)	0.04 (0.01, 0.08) *
**Country**				
Asia	−0.17 (−0.32, −0.03) *	−0.24 (−0.43,−0.06) **	−0.27 (−0.45,−0.09) **	0.05 (−0.01, 0.11)
EU/N. America	−0.08 (−0.17, 0.01)	−0.04 (−0.15, 0.07)	−0.04 (−0.13, 0.06)	0.04 (−0.00, 0.09)
**BMI**				
Normal	−0.06 (−0.16, 0.00)	−0.05 (−0.18, 0.08)	−0.02 (−0.13, 0.09)	0.04 (−0.01, 0.09)
Overweight	−0.20 (−0.40, 0.01)	−0.07 (−0.31, 0.16)	−0.08 (−0.29, 0.13)	0.03 (−0.61, 0.12)
Mixed	−0.14 (−0.27, −0.00) *	−0.23 (−0.40, −0.05) *	−0.28 (−0.46, −0.10) **	0.07 (0.01, 0.14) *
**Disease**				
With disease	−0.20 (−0.43, 0.04)	−0.13 (−0.33, 0.07)	−0.20 (−0.38, −0.02) *	0.06 (−0.01, 0.12)
No disease	−0.08 (−0.17, 0.00)	−0.08 (−0.20, 0.02)	−0.05 (−0.15, 0.05)	0.05 (0.00, 0.09) *
**Baseline levels**				
Normal	−0.21 (−0.43, 0.02)	−0.08 (−0.19, 0.04)	−0.08 (−0.18, 0.01)	0.04 (−0.01, 0.09)
Dyslipidemia	−0.09 (−0.18, −0.01) *	−0.10 (−0.32, 0.12)	−0.1 (−0.30, 0.08)	0.05 (0.00, 0.10) *

^1^ Significance levels: ** for *p* value ≤ 0.01, * for *p* value ≤ 0.05. See Supplementary Materials Table S2 for complete data (DM, 95% CI, *p* value, *I*^2^ and *p* value for Q test).

**Table 4:** Subgroup analysis: stratification by type of flavonol and dose of intervention.

Factor Subgroups	TAG (mmol/L) DM (95% CI) ^1^	TC (mmol/L) DM (95% CI) ^1^	LDL (mmol/L) DM (95% CI) ^1^	HDL (mmol/L) DM (95% CI) ^1^
**Compound**				
Pure	−0.17 (−0.35, 0.00) *	−0.12 (−0.31, 0.06)	−0.18 (−0.34, 0.02) *	0.06 (−0.01, 0.12)
Mixture	−0.10 (−0.18, −0.04) *	−0.09 (−0.20, 0.02)	−0.06 (−0.16, 0.04)	0.04 (−0.00, 0.08)
**Dose**				
>200 (mg/day)	−0.22 (−0.18, 0.00)	−0.20 (−0.43, 0.03)	−0.27 (−0.48, −0.06) *	0.06 (−0.02, 0.13)
<200 (mg/day)	−0.10 (−0.18, −0.02) *	−0.08 (−0.18, 0.03)	−0.05 (−0.15, 0.04)	0.04 (−0.00, 0.08) *

^1^ Significance levels: * for *p* value ≤ 0.05. See Supplementary Materials Table S2 for complete data (DM, 95% CI, *p* value, *I*^2^ and *p* value for Q test).

**Table 5:** Summary for effect of flavonol supplementation on the measured biomarkers and quality of evidence.

Biomarker	*n* (N_T_/N_C_)	DM (95% CI), *p* Value	GRADE ^1^
TAG (mmol/L)	17 (467/456)	−0.10 (−0.18; −0.03) *p* = 0.007	Low ^2,3^
TC (mmol/L)	18 (473/462)	−0.10 (−0.20; −0.01) *p* = 0.023	Moderate ^2^
LDL (mmol/L)	18 (473/462)	−0.14 (−0.21; −0.07) *p* = 0.000	Moderate ^2^
HDL (mmol/L)	18 (473/462)	0.05 (0.02; 0.07) *p* = 0.000	Moderate ^2^
SBP (mmHg)	16 (370/362)	−4.84 (−5.64; −4.04) *p* = 0.000	Low ^2,3^
DBP (mmHg)	16 (370/362)	−3.32 (−4.09; −2.55) *p* = 0.000	Low ^2,3^
Glucose (mmol/L)	12 (276/270)	−0.18 (−0.29; −0.07) *p* = 0.001	Moderate ^2^

*n*, number of studies; N_T_, number of supplemented participants, N_C_, number of control participants; DM, difference in means, CI, 95% confidence interval. ^1^ Quality of evidence was based on the GRADE system [28]. It was downgraded from high to moderate in the presence of either serious risk of bias across studies or serious risk of reporting bias, and downgraded to low if both were present. No further downgrade was necessary because there was no evidence of heterogeneity, no indirectness of evidence and no imprecision of results; ^2^ Serious risk of bias across studies: more than 50% of the studies had unclear allocation concealment, many of the studies were single blinded instead of double blinded and account of participant losses was also incomplete in many studies; ^3^ Serious risk of reporting bias: Eggerts *p*-value was lower than 0.05 and a considerable proportion of small studies was present (31% to 42% of studies with 12 to 24 participants).

## Supplementary Materials

The following are available online at http://www.mdpi.com/2072-6643/9/2/117/s1, Figure S1: Funnel plots and Eggers statistics for: lipids (a); blood pressure (b); and glucose (c), Table S1: Quality assessment of selected studies, Table S2: Subgroup analysis, Table S3: Sensitivity analysis in country, disease status and baseline levels.
