# Supplementary material for: Impact of Flavonols on Cardiometabolic Biomarkers: A Meta-Analysis of Randomized Controlled Human Trials to Explore the Role of Inter-Individual Variability
Source: Nutrients. 2017 Feb 9;9(2):117. doi: 10.3390/nu9020117 (PMC5331548; doi:10.3390/nu9020117)
Supplement: Supplementary file 1 [file nutrients-09-00117-s001.docx]

Supplementary Material: A Meta-Analysis of Randomized Controlled Human Trials to Explore the Role of Inter-Individual Variability

Regina Menezes, Ana Rodriguez-Mateos, Antonia Kaltsatou, Antonio González-Sarrías, Arno Greyling, Christoforos Giannaki, Cristina Andres-Lacueva, Dragan Milenkovic, Eileen R. Gibney, Julie Dumont, Manuel Schär, Mar Garcia-Aloy, Susana Alejandra Palma-Duran, Tatjana Ruskovska, Viktorija Maksimova, Emilie Combet and Paula Pinto

**Table S1.** Quality assessment of selected studies.

| **Reference** | **Random Sequence Generation (Score)** | **Allocation Concealment (Score)** | **Clear Blinding (Score)** | **Compliance Measurment (Score)** | **Flow of Participants (Score)** | **Funding (Score)** | **Baseline Comparability (Score)** | **Data Report (Score) (Notes)** | **Risk of Bias ^1^ (Score)** |
| --- | --- | --- | --- | --- | --- | --- | --- | --- | --- |
| Brull et al*.* 2015 [31] | Yes (1) | Yes (1) | Participants  Researchers  Statitians (3) | Plasma quercetin; capsule count (1) | Explained in detail (1) | Government (1) | Yes (1) | Complete (1) | Low (10) |
| Chen et al. 2015 [39] | Yes (1) | Yes (1) | Participants  Researchers  Statitians (3) | Self-reported (0.5) | Explained in detail (1) | Government (1) | Yes(1) | Complete (1) | Low (9.5) |
| Choi et al. 2015 [43] | Yes (1) | Unclear (0) | Participants  Researchers (2) | NR (0) | NR (0) | Government (1) | Yes (1) | Complete (1) | Moderate (6) |
| Chopra et al. 2000 [32] | Yes (1) | Unclear (0) | Participants (1) | Self-reported (0.5) | Unclear (0) | Company (0) | Yes (1) | Incomplete (0) (Values for pre-treatment are the post-placebo values) | High (3.5) |
| Conquer et al. 1998 [38] | Yes (1) | Unclear (0) | Participants  Researchers (2) | Plasma quercetin; capsule count (1) | NR (0) | Government (1) | Yes (1) | Complete (1) | Moderate (7) |
| Dower et al. 2015 [33] | Yes (1) | Yes (1) | Participants  Researchers  Statitians (3) | Plasma flavonoids; self-report (1) | Explained in detail (1) | Public-private; author from company (0) | Yes (1) | Complete (1) | Low (9) |
| Edwards et al. 2007 [37] | Yes (1) | Unclear (0) | Participants  Researchers (2) | Plasma quercetin; capsule count (1) | Explained in detail (1) | University (1) | Unclear (0) | Complete (1) | Moderate (7) |
| Egert et al. 2009 [29] | Yes (1) | Unclear (0) | Participants  Researchers (2) | Plasma quercetin; capsule count (1) | Explained in detail (1) | Government (1) | Yes (1) | Complete (1) | Low (8) |
| Javadi et al. 2014 [46] | Yes (1) | Unclear (0) | Participants (1) | NR (0) | Explained in detail (1) | University (1) | Yes (1) | Complete (1) | Moderate (6) |
| Kim et al. 2013 [42] | Yes (1) | Unclear (0) | Participants (1) | Self-reported (0.5) | NR (0) | Government (1) | Yes (1) | Complete (1) Data for t-Chol obtained from graphic | Moderate (5.5) |
| Kim et al. 2015 [44] | Yes (1) | Unclear (0) | Participants (1) | NR (0) | NR (0) | University (1) | Yes (1) | Complete (1) | Moderate (5) |
| Larmo et al. 2009 [35] | Yes (1) | Yes (1) | Participants  Researchers  Statitians (3) | Plasma quercetin; self-reported (1) | Explained in detail (1) | University (1) | Yes (1) | Complete (1) | Low (10) |
| Lee et al. 2011 [41] | Yes (1) | Unclear (0) | Participants (1) | Self-reported (0.5) | NR (0) | NR (0) | No (0) | Complete (1) | High (3.5) |
| Lu et al. 2015 [40] | Yes (1) | Unclear (0) | Participants (1) | Plasma total phenolics (1) | Explained in detail (1) | NR (0) | Yes (1) | Complete (1) | Moderate (6) |
| Pfeuffer et al. 2013 [30] | Yes (1) | Unclear (0) | Participants (1) | Plasma quercetin; capsule count (1) | Explained in detail (1) | Government (1) | Yes (1) | Incomplete (0) No pre values for placebo and treatment; only baseline values | Moderate (6) |
| Shi & Williamson 2016 [36] | Yes (1) | Yes (1) | Participants  Researchers (2) | Plasma quercetin; capsule count (1) | Explained in detail (1) | Government & University (1) | Yes (1) | Complete (1) | Low (9) |
| Suomela et al. 2006 [34] | Yes (1) | Unclear (0) | Participants (1) | NR (0) | NR (0) | Government (1) | Yes (1) | Complete (1) | Moderate (5) |
| Zahedi et al. 2013 [45] | Yes (1) | Yes (1) | Participants  Researchers (2) | Capsule count(0.5) | Explained in detail (1) | University (1) | Yes (1) | Incomplete (0) BP data not correctly reported | Moderate (7.5) |

^1^ Low risk of bias: 8 to 10; moderate risk of bias: 5 to 7.5; High risk of bias: <5.

**Table S2.** Subgroup analyses: stratification by participants’ characteristics and study characteristics.

| **Outcome** | | **TAG** | | | | **TC** | | | |
| --- | --- | --- | --- | --- | --- | --- | --- | --- | --- |
| Factor | Subgroups | *N/n* | DM (95%CI) | *P* value(*I^2^*)^1^ | *Q* test ^2^ | *N/n* | DM (95%CI) | *P* value (*I^2^*) ^1^ | *Q* test ^2^ |
| Age | Above 40 | 4/111 | −0.111(-0.351, 0.130) | 0.368 (0%) | 0.974 | 4/111 | −0.026 (−0.409, 0.358) | 0.896 (0%) | 0.890 |
|  | Mixed ages | 11/700 | −0.106 (−0.188, −0.025) | 0.011 (0%) |  |  |  |  |  |
|  |  |  |  |  |  | 11/700 | −0.095 (−0.195, −0.003) | 0.062 (0%) |  |
| Sex | Female | 2/99 | −0.145 (−0.402, 0.111) | 0.266 (0%) | 0.939 | 3/111 | −0.221 (−0.529, 0.086) | 0.159 (0%) | 0.654 |
|  | Male | 3/63 | −0.105 (−0.424, 0.215) | 0.520 (0%) |  | 3/63 | −0.028 (−0.448, 0.392) | 0.895 (0%) |  |
|  | Mixed sexes | 10/649 | −0.103 (−0.187, −0.019) | 0.016 (0%) |  | 10/649 | −0.084 (−0.187, 0.019) | 0.111 (0%) |  |
| Country | Asia | 5/255 | −0.175 (−0.319, −0.032) | 0.017 (0%) | 0.266 | 6/267 | −0.244 (−0.429, −0.059) | 0.010 (0%) | 0.069 |
|  | EU & N. America | 10/556 | −0.079(−0.170, 0.013) | 0.092 (0%) |  | 10/556 | −0.044 (−0.155, 0.066) | 0.429 (0%) |  |
| BMI | Mixed BMI | 9/338 | −0.140 (−0.275, −0.004) | 0.043 (0%) | 0.628 | 9/338 | −0.228 (−0.403, −0.054) | 0.010 (0%) | 0.254 |
|  | Normal | 1/229 | −0.060 (−0.165, 0.0045) | 0.262 (0%) |  | 1/229 | −0.049 (−0.175, 0.077) | 0.448 (0%) |  |
|  | Overweight | 5/274 | −0.197 (−0.403, 0.008) | 0.060 (0%) |  | 5/274 | −0.074 (−0.313, 0.165) | 0.543 (0%) |  |
| Disease status | Disease | 5/305 | −0.196 (−0.431, 0.038) | 0.100 (0%) | 0.384 | 5/305 | −0.131 (−0.331, 0.070) | 0.202 (0%) | 0.725 |
|  | No disease | 8/399 | −0.085 (−0.174, 0.003) | 0.054 (0%) |  | 9/411 | −0.084 (−0.203, 0.024) | 0.124 (0%) |  |
| Baseline levels | Dyslipidaemia | 6/324 | −0.207 (−0.433, 0.019) | 0.072 (0%) | 0.415 | 7/283 | −0.100 (−0.316, 0.116) | 0.363 (0%) | 0.664 |
|  | Normal | 9/487 | −0.093 (−0.176, -0.010) | 0.026 (0%) |  | 6/439 | −0.075 (−0.188, 0.039) | 0.198(0%) |  |
| Type of flavonol | Mixture | 7/471 | −0.091 (−0.177, 0.045) | 0.039 (0%) | 0.411 | 8/493 | −0.087 (−0.197, 0.024) | 0.123 (0%) | 0.727 |
|  | Pure | 8/340 | −0.172 (−0.347, 0.000) | 0.053 (0%) |  | 8/340 | −0.125 (−0.310, 0.060) | 0.185 (0%) |  |
| Dose (mag/day) | Above 200 | 5/190 | −0.215 (−0.177, 0.004) | 0.127 (0%) | 0.424 | 5/190 | −0.202 (−0.433, 0.030) | 0.087 (0%) | 0.329 |
|  | Below 200 | 10/621 | −0.098 (−0.178, −0.017) | 0.018 (0%) |  | 11/633 | −0.076 (−0.179, 0.028) | 0.0153 (0%) |  |

^1^ p value for intervention effect and inconsistency within subgroup. ^2^ p value for heterogeneity between subgroups. N= number of studies; n= number of participants; DM, difference in means; CI, 95% confidence interval. Subgroup analysis was performed after removal of studies with high risk of bias.

**Table S2** (continuation). Subgroup analyses: stratification by participants’ characteristics and study characteristics.

| **Outcome** | | **LDL** | | | | **HDL** | | | |
| --- | --- | --- | --- | --- | --- | --- | --- | --- | --- |
| Factor | Subgroups | *N/n* | DM (95%CI) | *P* value (*I*^2^) ^1^ | *Q* test ^2^ | *N/n* | DM (95%CI) | *P* value (*I*^2^) ^1^ | *Q* test ^2^ |
| Age | Above 40 | 4/111 | −0.078 (−0.376, 0.221) | 0.610 (0%) | 0.851 | 4/111 | 0.047 (−0.096, 0.190) | 0.520 (0%) | 0.981 |
|  | Mixed ages | 11/700 | −0.091 (−0.181, −0.002) | 0.045 (0%) |  | 11/700 | 0.045 (0.008, 0.082) | 0.017 (0%) |  |
| Sex | Female | 2/99 | −0.122 (−0.413, −0.168) | 0.409 (0%) | 0.972 | 2/99 | 0.043 (−0.057, 0.143) | 0.403 (0%) | 0.966 |
|  | Male | 3/63 | −0.075 (−0.406, 0.256) | 0.658 (0%) |  | 3/63 | 0.067 (−0.101, 0.235) | 0.432 (0%) |  |
|  | Mixed sexes | 10/649 | −0.088 (−0.181, 0.005) | 0.063 (12.7%) |  | 10/649 | 0.045 (0.005, 0.084) | 0.028 (0%) |  |
| Country | Asia | 5/255 | −0.274 (−0.455, −0.093) | 0.003 (26%) | 0.024 | 5/255 | 0.051 (−0.010, 0.112) | 0.103 (0%) | 0.829 |
|  | EU & N. America | 10/556 | −0.037 (−0.134, 0.060) | 0.451 (0%) |  | 10/556 | 0.042 (−0.002, 0.087) | 0.061 (0%) |  |
| BMI | Mixed BMI | 8/246 | −0.282 (−0.462, −0.102) | 0.002 (0%) | 0.056 | 8/246 | 0.073 (0.006, 0.141) | 0.034 (0%) | 0.669 |
|  | Normal | 1/229 | −0.020 (−0.135, 0.095) | 0.734 (0%) |  | 1/229 | 0.040 (−0.012, 0.092) | 0.130 (0%) |  |
|  | Overweight | 5/274 | −0.081 (−0.293, 0.132) | 0.457 (0%) |  | 5/274 | 0.029 (−0.61, 0.119) | 0.524 (0%) |  |
| Disease status | Disease | 5/305 | −0.201(−0.378, −0.024) | 0.026 (28.4%) | 0.153 | 5/305 | 0.056 (−0.009, 0.122) | 0.093 (0%) | 0.802 |
|  | No disease | 8/399 | −0.052 (−0.155, 0.052) | 0.327 (0%) |  | 8/399 | 0.046 (0.000, 0.092) | 0.049 (0%) |  |
| Baseline levels | Dyslipidaemia | 7/283 | −0.108 (−0.295, 0.080) | 0.261 (0%) | 0.837 | 12/520 | 0.052 (0.000, 0.103) | 0.049 (0%) | 0.773 |
|  | Normal | 7/456 | −0.085 (−0.185, 0.014) | 0.092 (33.7%) |  | 2/256 | 0.041 (−0.011, 0.092) | 0.120 (0%) |  |
| Type of flavonol | Mixture | 5/471 | −0.056 (−0.157, 0.045) | 0.281 (0%) | 0.208 | 5/471 | 0.040 (−0.004, 0.083) | 0.073 (0%) | 0.662 |
|  | Pure | 8/340 | −0.177 (−0.338, 0.017) | 0.053 (0%) |  | 8/340 | -0.057 (−0.007, 0.121) | 0.079 (0%) |  |
| Dose (mag/day) | Above 200 | 5/190 | −0.266 (−0.476, −0.056) | 0.013 (6.2%) | 0.072 | 5/190 | 0.060 (−0.015, 0.135) | 0.116 (0%) | 0.660 |
|  | Below 200 | 10/621 | −0.055 (−0.149, 0.039) | 0.249 (0%) |  | 11/633 | 0.041 (−0.000, 0.082) | 0.050 (0%) |  |

^1^ p value for intervention effect and inconsistency within subgroup. ^2^ p value for heterogeneity between subgroups. *N* = number of studies; *n*= number of participants; DM, difference in means; CI, 95% confidence interval. Subgroup analysis was performed after removal of studies with high risk of bias.

**Table S3.** Sensitivity analysis in country, disease status and baseline levels.

| **Outcome** | | **TAG** | | | | **TC** | | | |
| --- | --- | --- | --- | --- | --- | --- | --- | --- | --- |
| Factor | Subgroups ^1^ | *N/n* | DM (95%CI) | *p* value (*I*^2^) ^2^ | *Q* test ^3^ | *N/n* | DM (95%CI) | *p* value (*I^2^*)^2^ | *Q* test ^3^ |
| Country | Asia | 3/133 | −0.168 (−0.329, −0.006) | 0.042 (0%) | 0.311 | 4/145 | −0.285 (−0.544, −0.027) | 0.030 (0%) | 0.098 |
|  | EU & N. America | 7/373 | −0.071 (−0.166, 0.024) | 0.144 (0%) |  | 7/373 | −0.046 (−0.164, 0.072) | 0.449 (0%) |  |
| Disease status | Disease | 4/243 | −0.201(−0.455, 0.053 ) | 0.121 (0%) | 0.341 | 4/243 | −0.135 (−0.355, 0.082) | 0.231 (0%) | 0.498 |
|  | No disease | 6/338 | −0.069 (−0.167, 0.030) | 0.171 (0%) |  | 6/338 | −0.048 (−0.169, 0.073) | 0.438 (0%) |  |
| Baseline levels | Dyslipidaemia | 5/262 | −0.213 (−0.457, 0.030) | 0.087 (0%) | 0.275 | 6/259 | −0.020 (−0.253, 0.213) | 0.867 (0%) | 0.472 |
|  | Normal | 6/354 | −0.067 (−0.163, 0.028) | 0.188 (0%) |  | 2/256 | −0.046 (−0.174, 0.082) | 0.480 (0%) |  |
| Outcome | | LDL | | | | HDL | | | |
| Country | Asia | 3/133 | −0.239 (−0.524, 0.047) | 0.101 (0%) | 0.182 | 3/133 | 0.036 (−0.061, 0.132) | 0.465 (0%) | 0.915 |
|  | EU & N. America | 7/373 | −0.032 (−0.136, 0.072) | 0.546 (0%) |  | 7/373 | 0.042 (−0.006, 0.090) | 0.088 (0%) |  |
| Disease status | Disease | 4/243 | −0.257(−0.463, −0.052 ) | 0.014 (33%) | 0.053 | 4/243 | 0.072 (−0.004, 0.147) | 0.064 (0%) | 0.537 |
|  | No disease | 6/338 | −0.028 (−0.135, 0.079) | 0.603 (0%) |  | 6/338 | 0.043 (−0.006, 0.092) | 0.083 (0%) |  |
| Baseline levels | Dyslipidaemia | 6/259 | −0.070 (−0.265, 0.125) | 0.483 (0%) | 0.925 | 8/325 | 0.071 (0.002, 0.139) | 0.042 (0%) | 0.737 |
|  | Normal | 5/357 | −0.080 (−0.186, 0.025) | 0.135 (51.7%) |  | 2/256 | 0.041 (−0.011, 0.092) | 0.120 (0%) |  |

^1^ Analysis of country subgroups were performed after removal of studies with high risk of bias and studies conducted with participants with diagnosed diseases; analysis of disease status and baseline subgroups were performed after removal of studies with high risk of bias and studies with Asian countries; ^2^ *p* value for intervention effect and inconsistency within subgroup; ^3^ p value for heterogeneity between subgroups; *N*= number of studies; n= number of participants; DM, difference in means; CI, 95% confidence interval.


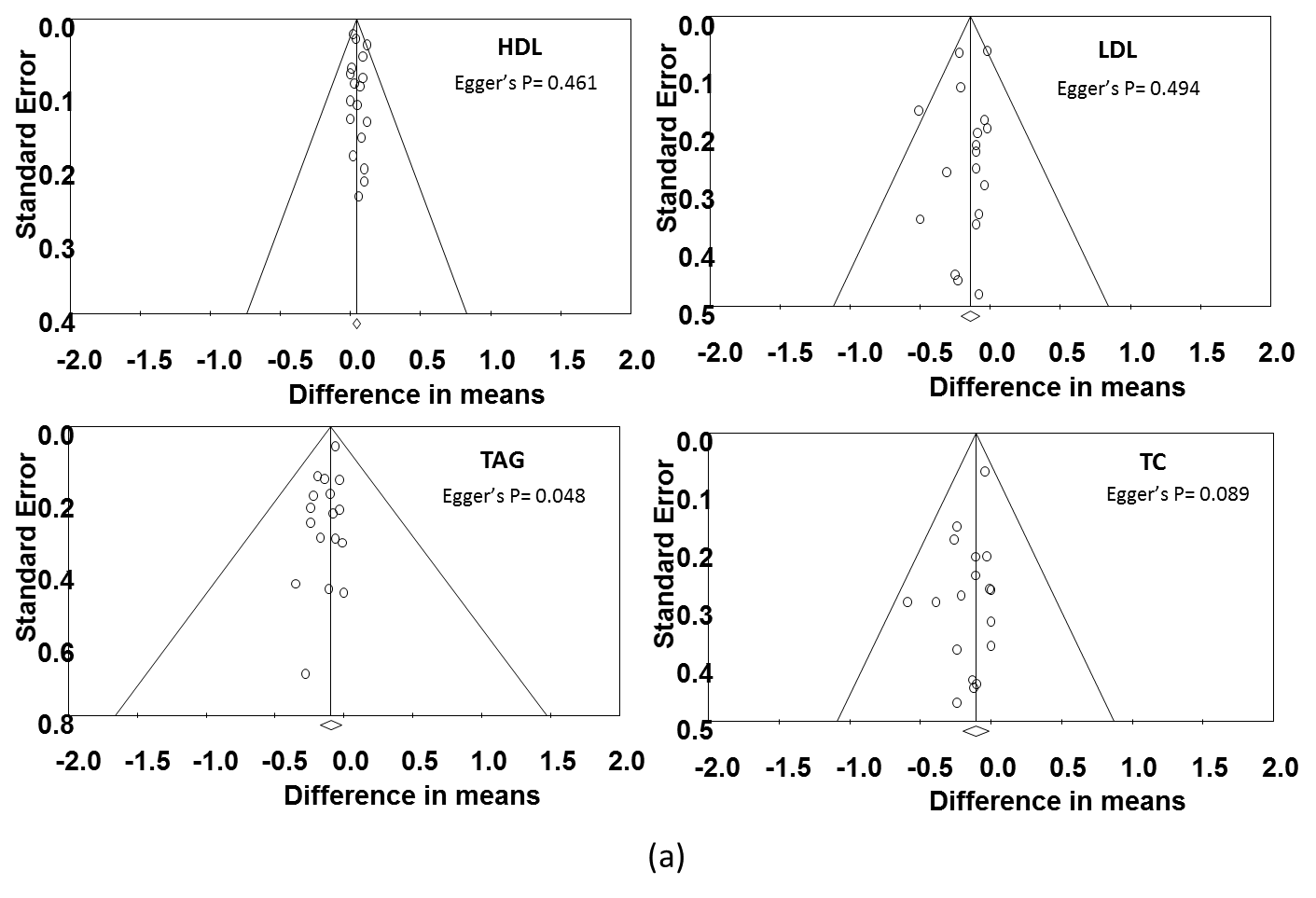


| 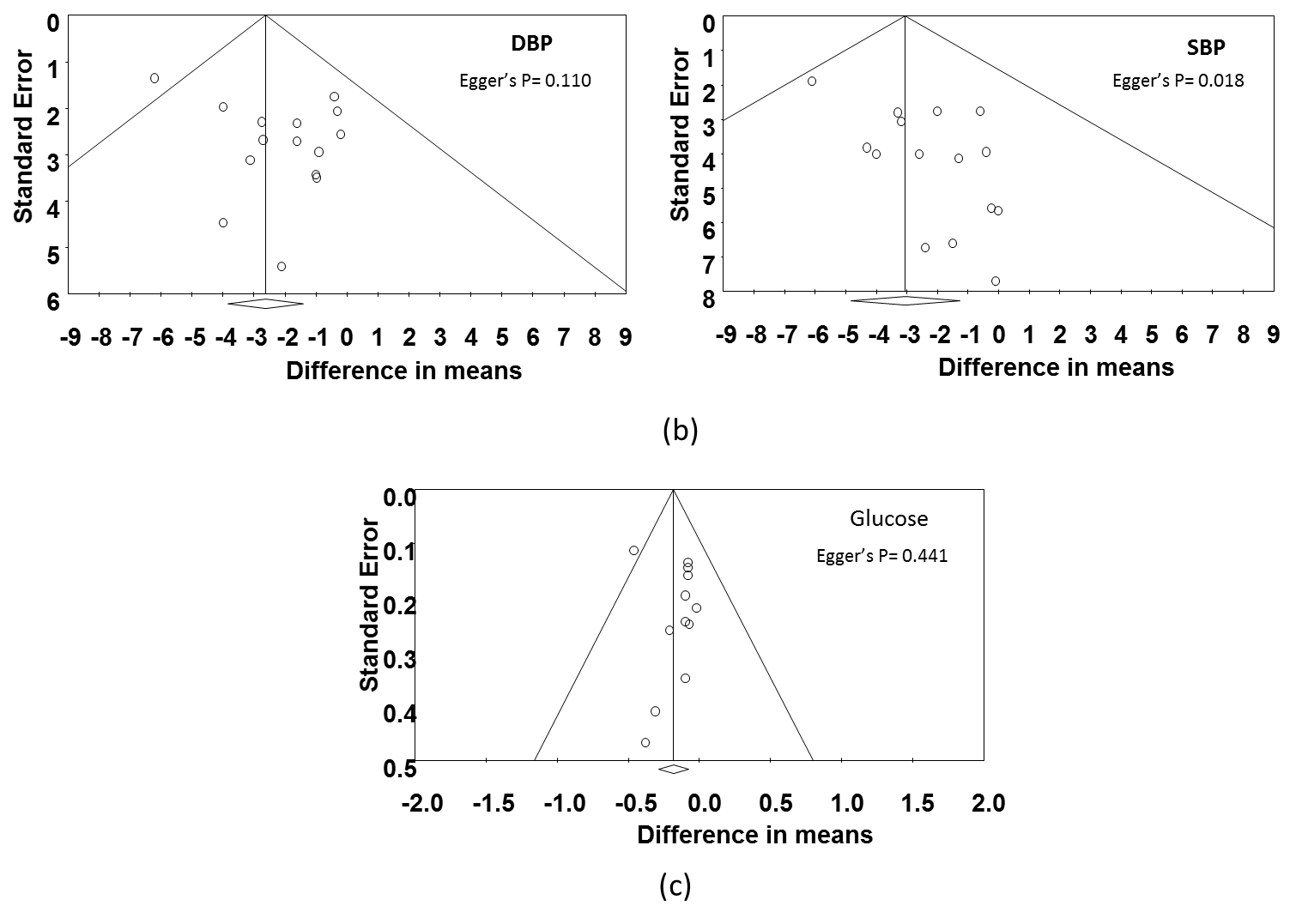 |
| --- |
|  |

**Figure S1.** Funnel plots and Eggers statistics for lipids (**a**), blood pressure (**b**) and glucose (**c**).
